# Synthesis of small Ni-core–Au-shell catalytic nanoparticles on TiO_2_ by galvanic replacement reaction†‡

**DOI:** 10.1039/d0na00617c

**Published:** 2021-01-08

**Authors:** Julien Reboul, Z. Y. Li, Jun Yuan, Kazuki Nakatsuka, Masakazu Saito, Kohsuke Mori, Hiromi Yamashita, Yu Xia, Catherine Louis

**Affiliations:** Sorbonne Université, CNRS, Laboratoire de Réactivité de Surface (LRS) Paris F-75005 France Catherine.louis@upmc.fr; School of Physics and Astronomy, University of Birmingham Birmingham B15 2TT UK; Department of Physics, University of York Heslington York YO10 5DD UK; Division of Materials and Manufacturing Science, Graduate School of Engineering, Osaka University 2-1 Yamada-oka Suita Osaka 565-0871 Japan; Department of Chemistry and Materials Science, National Institute of Technology, Gunma College 580 Toriba-machi Maebashi Gunma 371-8530 Japan; Department of Materials Science and Engineering, Southern University of Science and Technology Shenzhen Guangdong 518055 China

## Abstract

We report the first preparation of small gold–nickel (AuNi) bimetallic nanoparticles (<5 nm) supported on titania by the method of galvanic replacement reaction (GRR), evidenced by the replacement of Ni atoms by Au atoms according to the stoichiometry of the reaction. We showed that this preparation method allowed not only the control of the gold and nickel contents in the samples, but also the formation of small bimetallic nanoparticles with strained core–shell structures, as revealed by aberration-corrected scanning transmission electron microscopy in combination with energy-dispersive X-ray spectroscopy mapping. The catalytic characterization by the probe reaction of semi-hydrogenation of butadiene showed that the resulting nickel-based nanocatalysts containing a small amount of gold exhibited higher selectivity to butenes than pure nickel catalysts and a high level of activity, closer to that of pure nickel catalysts than to that of pure gold catalysts. These improved catalytic performances could not be explained by a mere structural model of simple core–shell structure of the nanoparticles. Instead, they could come from the incorporation of Ni within the gold surface and/or from surface lattice relaxation and subsurface misfit defects.

## Abstract 2

Galvanic replacement reaction to synthesize small catalytic bimetallic Au–Ni nanoparticles with Ni core–Au shell structure directly on an oxide support.

## Graphical abstract

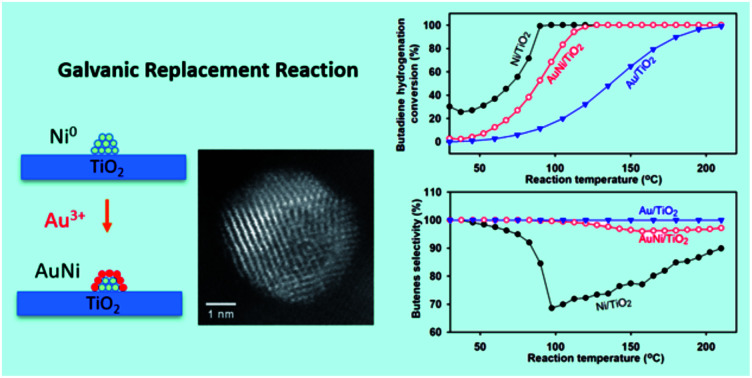

## Introduction

Supported bimetallic gold–nickel (AuNi) nanoparticles form an interesting catalytic system for several types of reactions, such as hydrodechlorination of 2,4-dichlorophenol^1,2^ and chlorobenzene,^3^ chemoselective hydrogenation of nitroarenes,^4–6^ selective hydrogenation of alkynes or dienes^7–9^ and of levulinic acid,^10^ methanation of syngas^11^ and steam reforming of light hydrocarbons.^12,13^ The phase diagram of binary Au–Ni system has a large domain in which Ni and Au are not miscible (so called the miscibility gap), excluding any AuNi alloy formation at low temperatures (1 : 1 alloy forms only for *T* > 740 °C).^14^ However, according to the thermodynamic calculations of Xiong *et al.*,^15^ it is in principle possible to synthesize small bimetallic alloy-type AuNi particles at the nanoscale as the miscibility gap becomes smaller (and even disappears) with the decrease of the particle size. This size effect has been recently demonstrated experimentally by Bogatyrenko *et al.*^16^

Using classical methods of preparation of supported metal catalysts based on the principle of deposition–reduction, *i.e.*, deposition of a metal precursor onto a support followed by thermal reduction, several papers show that it is difficult to get bimetallic AuNi nanoparticles (NPs) with a composition close to the nominal one and/or with a homogeneous composition.^2,8,9,13,17–19^ Another strategy for the preparation of supported AuNi catalysts is the reduction–deposition approach, which involves the synthesis of colloidal bimetallic NPs followed by their deposition onto a support. Colloid synthesis requires stabilizing agents and reducing agents. It can be performed in various media: water,^20,21^ organic solvent^22–29^ or biphasic medium (in the case of reverse emulsions),^30–32^ and involves either single- or two-step preparations. These preparations lead to various forms of AuNi NPs: alloy, AuNi or NiAu core–shell or dumbbell. The deposition of the aforementioned NPs onto a support can then be achieved by impregnation or better, by electrostatic adsorption.^23,31,32^ The advantage of reduction–deposition over deposition–reduction is that the NPs are more homogeneous in size and composition, and that several NP structures can be obtained as mentioned above.^20,21,30–32^ However, the reduction–deposition method also presents several disadvantages, and specifically in the case of catalytic applications. (i) The reducing agent can contaminate the NPs, hence impact the catalytic performances as pointed out in a number of papers.^20,33,34^ (ii) The chemical agents stabilizing the NPs must be eliminated prior to the catalytic reaction, specifically in the case of gas phase reaction. In most cases, the decomposition of those stabilizing agents is carried out with an additional calcination step. However, these thermal treatments can be detrimental to both the original size distribution and structures of the NPs.^23,35^

Galvanic replacement reaction (GRR), also called galvanic displacement or direct redox reaction, is an elegant way to prepare supported bimetallic NPs.^36,37^ Galvanic replacement of metal 1 (M1) by metal 2 (M2) leads to the deposition of metal 2 onto metal 1, provided that the redox potential of M2 ions (Ox2) is higher than that of M1 ions (Ox1), so that the following reaction can take place:

1 Ox2 + Red1 → Ox1 + Red2

M2 ions in aqueous solution reduce onto the surface of metal 1 (Red1) while M1 ions are released into solution (Red2). According to this equation, the amount of M2 deposited cannot exceed that of M1.

This technique was first developed in metallurgy to coat or plate metal surfaces by another metal. It is also often used in electrochemistry^38–42^ and in metallization of semiconductors.^36^ In addition, GRR has been employed to prepare bimetallic colloids^43–46^ and hollow NPs or nanocages. Galvanic replacement of Ag by Au, Pt and Pd leads to the formation of Au, Pt and Pd nanocages, respectively.^45^ GRR has also been used to prepare supported bimetallic NPs, for instance Au–Ag, Au–Pd, Au–Cu supported on various types of supports.^47–54^ In this case, galvanic replacement is performed after the precursor of the first metal is deposited and reduced as metal nanoparticles on a support. Bimetallic NPs can be obtained whether the metals are miscible, for instance Au–Ag, Au–Cu, and Au–Pd *etc.* or immiscible, such as Au–Pt. In addition, it can also directly produce M1-core-M2-shell NPs.

GRR is applicable to the preparation of supported Au–Ni catalysts because the standard redox potential of Au^3+^ is much higher (+1.0 V *vs.* SHE (standard hydrogen electrode) for Au^III^Cl_4_^−^/Au) than that of Ni^2+^ (−0.26 V *vs.* SHE for Ni^2+^/Ni^0^) in the following reaction:

2 2Au^3+^ + 3Ni^0^ → 2Au^0^ + 3Ni^2+^

There are examples in the literature^55–59^ demonstrating the use of GRR to coat nickel surfaces by gold. It also has been shown^60–62^ that AuNi colloids can be prepared with this method, all resulting in the formation of nickel particles decorated or covered by gold. So far, the closest examples of GRR applied to the preparation of supported Au–Ni catalysts come from three teams: the group of Keane *et al.*^4,63^ prepared AuNi/Al_2_O_3_ catalysts by first impregnating Al_2_O_3_ with Ni(NO_3_)_2_, followed by a thermal reduction step, before exposing Ni/Al_2_O_3_ to a HAuCl_4_ solution (Au/Ni = 0.1 and 10). The preparative route was termed by the authors as a direct redox method despite performing an intermediate step of passivation of the supported Ni^0^ particles achieved with O_2_ diluted in He at room temperature (RT) prior to contact with the HAuCl_4_ solution. Wu *et al.*^3^ prepared AuNi/TiO_2_ (Au/Ni = 0.03 and 0.1) according to the same principle, except that after Ni reduction, Ni^0^/TiO_2_ was introduced in the solution of HAuCl_4_ without air contact. In both procedures, the initial Ni particles were very large (>30 nm), so were the resulting AuNi particles. Smaller AuNi nanoparticles displaying a bimodal size distribution (with sizes below 5 nm and a Au/Ni atomic ratio of 0.06) were recently synthesized by Wang *et al.* by adding a HAuCl_4_ solution to small Ni^0^ NPs (the size was not reported) supported on Ni–Mg–Al hydrotalcite (HT). In this work, the Ni^0^ NPs were obtained by reduction of Ni^2+^ present in the structure of the hydrotalcite with NaBH_4_ at RT.^64^ The formation of homogeneously alloyed structures was proposed, based on scanning transmission electron microscopy–energy dispersive X-ray (STEM–EDX) measurement of a relatively large particle of around 6 nm. However, the work was conducted at low spatial resolution and e-beam damages were also observed during the scan. The paper did not make clear how representative this result was. To sum up, the current literature about GRR-related processes for the synthesis of supported AuNi NPs consists of only a few examples dealing with the formation of either large AuNi NPs by an unconventional GRR process (in which a Ni^0^-passivation step was applied before adding the Au source) or small AuNi NPs with limited characterization of the structure and the chemical composition.

The purpose of this paper was to validate the GRR technique in the scope of the preparation of small supported bimetallic AuNi nanoparticles, and to unambiguously determine the bimetallic character as well as the structure of the particles. Hence, the GRR technique was applied to prepare catalysts consisting of AuNi particles with size below 5 nm, supported on TiO_2_ and with low Au/Ni ratios (Au/Ni < 1). To achieve this goal, the method of deposition–precipitation has been chosen to prepare the initial Ni/TiO_2_ samples to achieve small Ni^0^ particles.^8^ The GRR process has been investigated using electron microscopy. The Au–Ni stoichiometry and the size of the resulting bimetallic NPs was monitored as a function of the nominal Au/Ni molar ratio. Electron microscopy was also used for structural characterization of individual bimetallic nanostructures. NP characterization was carried out on both as-prepared AuNi/TiO_2_ samples and samples obtained after a thermal treatment identical to the one carried out during the *in situ* activation of the catalysts prior to the reaction of selective hydrogenation. The reaction of selective hydrogenation of butadiene was used as an indirect characterization tool to probe the bimetallic character of the metal nanoparticles and the presence of Au–Ni interactions. The activity and selectivity of the AuNi/TiO_2_ samples were compared to those of the monometallic counterparts, namely Ni/TiO_2_ and Au/TiO_2_.

## Experimental

TiO_2_ was used as a support (from Evonik P25: 45 m^2^ g^−1^, nonporous, 70% anatase, 30% rutile, purity >99.5%). HAuCl_4_·3H_2_O and nickel nitrate hexahydrate, Ni(NO_3_)_2_·6H_2_O, were purchased from Aldrich, and urea was purchased from Sigma.

The monometallic nanoparticles of Ni metal on TiO_2_ (Ni/TiO_2_, 1.23 wt% of Ni) were prepared by deposition–precipitation with urea (DPU) in the absence of light, following previously reported procedure.^8^ Briefly, 61.07 mg (0.21 mmol) of Ni(NO_3_)_2_·6H_2_O and 1.26 g (21 mmol) of urea were dissolved in 50 mL of distilled water. Then, 1 g of pretreated TiO_2_ was added to the solution. The mixture solution was heated to 80 °C and maintained at the same temperature for 16 hours. After deposition–precipitation, the sample was collected by centrifugation and washed three times with distilled water. After drying at 80 °C for 2 hours, the as-synthesized Ni/TiO_2_ was stored at room temperature in a desiccator under vacuum.

The bimetallic AuNi/TiO_2_ samples were synthesized by galvanic replacement reaction. Before GRR, a reducing treatment was performed on the as-synthesized Ni/TiO_2_ to form Ni metal NPs on TiO_2_. 100 mg of as-synthesized Ni/TiO_2_ (21 μmol of Ni) was reduced by hydrogen flow at 350 °C for 2 hours in a reaction tube equipped with a closed septum. 10 mL of degassed distilled water was then added in the tube through the septum and the suspension was sonicated for 10 min. After sonication, a solution of gold precursor was added. Three different volumes of 1.189 mM HAuCl_4_ solution were added: 1.73, 3.46 and 5.19 mL, *i.e.*, 2.1, 4.1 and 6.2 μmol of Au, respectively, which corresponded to 10%, 20% and 30% of the initial atomic amount of Ni in Ni/TiO_2_. The suspensions were stirred for 1 hour at RT. Finally, the samples were recovered by centrifugation, washed three times with distilled water and dried under vacuum at RT. The as-prepared AuNi/TiO_2_ samples were stored at room temperature in a desiccator under vacuum. They were denoted Au(10)Ni/TiO_2_, Au(20)Ni/TiO_2_ and Au(30)Ni/TiO_2_ in the paper.

Metal loadings of the samples were determined by X-ray fluorescence (XRF) with a spectrometer XEPOS HE (AMETEK). The XRF analyses were calibrated using standard samples for each element.

Particle size measurements were performed by transmission electron microscopy (TEM) using a 200 kV JEOL 2010 at Sorbonne University. Bright field images were obtained with an Orius CCD camera (Gatan). Statistical analysis of the metal particles size in the reduced samples was obtained by counting around 300 particles, using the open source software ImageJ. The average particle diameter was deduced from the equation *d*_av_ = ∑*n*_*i*_*d*_*i*_/∑*n*_*i*_ where *n*_*i*_ was the number of the particles of diameter *d*_*i*_. The detection limit was ∼1 nm.

High angle annular dark field scanning transmission electron microscopy (HAADF-STEM) was performed at the University of Birmingham using a 200 kV JEOL 2100F electron microscope fitted with a CEOS probe corrector. The contrast of the resulting images depends strongly on the atomic number of the elements (*Z*-contrast) as well as the numbers of atoms interacting with the beam.^65,66^ A Bruker Xflash silicon drift detector was used to perform high-resolution elemental mapping within nanoparticles using Energy Dispersive X-ray Spectroscopy (EDX).

Hydrogenation of 1,3-butadiene was carried out with excess alkene to mimic the process of purification of an alkene C4 cut from highly unsaturated compounds in the same way as the previous studies on Au/TiO_2_ (ref. 67) and on AuNi/TiO_2_ prepared by co-deposition–precipitation.^8^ As in the previous studies, butene was replaced by propene for analytical reasons. The catalytic bed (200 mg) consisted of a few mg of catalyst diluted in SiC inserted in a Pyrex plug flow microreactor (4 mm of internal diameter). Before the reaction, the samples were activated *in situ* in an H_2_ flow (50 mL min^−1^) at 350 °C for 2 h, as Ni in Ni/TiO_2_ must be reduced to the metallic state for the reaction. According to TPR experiments performed with Ni/TiO_2_ also prepared by deposition–precipitation with urea,^8^ Ni is fully reduced at 350 °C. The same pretreatment was applied to the AuNi samples to avoid bias in the catalytic experiments. In order to highlight the influence of adding Au to Ni/TiO_2_, the catalytic beds contained roughly the same amount of nickel. The catalytic bed was then cooled to room temperature under H_2_ (for at least 30 min), and the gas mixture consisting of 0.3% butadiene, 30% propene and 20% hydrogen in He (49.7%) was introduced at RT with a total flow rate of 50 mL min^−1^, which corresponded to a space velocity of 30 L g^−1^ h^−1^ (or Gas Hourly Space Velocity (GHSV) = 20 000 h^−1^). The catalysts under the gas reaction mixture were heated at a rate of 0.5 °C min^−1^ up to 210 °C (300 °C for Au/TiO_2_). Gas analysis at the outlet of the reactor was performed every 15 min, *i.e.*, every 7.5 °C, from 30 °C to the final temperature by gas chromatography.

## Results

### Metal content

Given galvanic replacement reaction of Ni by Au, an increasing Au loading is expected in the samples, associated with a decreasing Ni loading with a factor of 1.5 due to the stoichiometry ratio of eqn (2). The theoretical as well as measured Ni and Au loadings in the AuNi/TiO_2_ samples determined by XRF are reported in Table 1. First, the Ni loading in the initial Ni/TiO_2_ sample is shown to be consistent with the nominal values, indicating that all Ni was deposited on TiO_2_ during deposition–precipitation with urea (DPU). As expected, the measured Au loading in the AuNi/TiO_2_ samples increased proportionally to the amounts of gold in solution. The Au loadings roughly corresponded to those expected although they were a little bit lower, suggesting that almost all gold was deposited onto the samples by GRR. Table 1 also shows that as expected from eqn (2), the Ni loading in AuNi/TiO_2_ decreased when the Au loading increased. Even though both the Au and Ni loadings were slightly lower than the theoretical values, the resulting Au/Ni atomic ratios corresponded to those expected; indeed, the experimental Au/Ni atomic ratios (0.13, 0.30, 0.55) were very similar to the theoretical Au/Ni atomic ratios calculated on the basis of eqn (2). These results provide a direct evidence for the galvanic replacement reaction. They also indicate the potential of GRR as a mean to finely tuning the Au/Ni atomic ratio.

**Table 1:** Theoretical and measured Ni and Au loadings and Au/Ni atomic ratios in the three AuNi/TiO_2_ samples and average metal particle size after GRR

Sample	Theoretical values			Experimental values			
	Ni^a^ (wt%)	Au (wt%)	Au/Ni at. ratio^a^	Ni (wt%)	Au (wt%)	Au/Ni at. ratio	particle size^b^ (nm)
Ni/TiO_2_	1.23	—	—	1.21	—	—	1.6 ± 0.5
Au(10)Ni/TiO_2_	1.05	0.41	0.12	0.75	0.33	0.13	3.7 ± 1.3
Au(20)Ni/TiO_2_	0.86	0.83	0.29	0.62	0.63	0.30	4.2 ± 1.1
Au(30)Ni/TiO_2_	0.68	1.24	0.55	0.58	1.07	0.55	4.7 ± 1.4
Au/TiO_2_^c^	—	1	—	—	1.20	—	2.7 ± 0.8

a Deduced from eqn (2).

b Average size deduced from TEM measurements.

c From ref. 8.

### Metal particle size

**Fig. 1:**
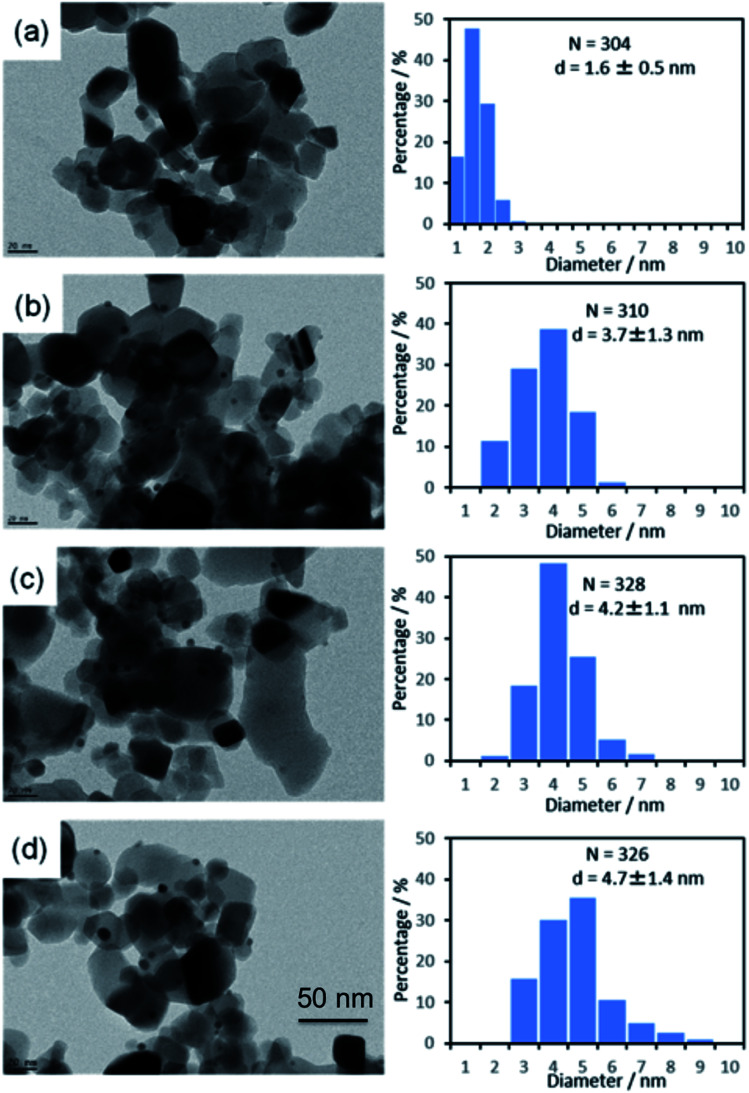
TEM images and particle size distributions of (a) Ni/TiO_2_ after reduction at 350 °C and as-prepared, (b) Au(10)Ni/TiO_2_, (c) Au(20)Ni/TiO_2_ and (d) Au(30)Ni/TiO_2_.

The average metal particle sizes in Ni/TiO_2_ (after thermal reduction at 350 °C in the same conditions as those of activation before the catalytic reaction, see Experimental) and in the three AuNi/TiO_2_ samples after GRR (as-prepared, without further thermal reduction) were determined from TEM images. Typical images of the samples are shown in Fig. 1, together with the size distributions and average metal particle sizes (also reported in Table 1) deduced from the measurement of around 300 particles. The first thing to note is that, as expected, the Ni particles in the monometallic Ni/TiO_2_ sample are very small with an average size of 1.6 nm. The second observation is that the AuNi NPs are larger than the Ni particles in Ni/TiO_2_, and their average size increases with the gold content. The latter observation is discussed in detail later.

### Reaction of hydrogenation of 1,3-butadiene

One way to probe the existence of interactions between Au and Ni in bimetallic particles is to test the AuNi samples in a selective catalytic reaction. The reaction selected herein was that of hydrogenation of 1,3-butadiene in the presence of an excess of propene as explained in the Experimental section.

The catalytic results obtained from Ni/TiO_2_, Au(10)Ni/TiO_2_, Au(20)Ni/TiO_2_, and Au(30)Ni/TiO_2_ are summarized in Table 2 together with those obtained from Au/TiO_2_ (1 wt%) studied in a previous paper under the same reaction condition^8^ and taken as a reference. The table reports the temperatures at which 100% conversion of butadiene was reached (*T*_100%_), which is an indication of the catalyst activity at 100% conversion. It can be seen that the Au/TiO_2_ sample is the least active catalyst with *T*_100%_ at the highest temperature (225 °C) despite the larger amount of gold atoms in the catalytic bed and the smaller sized AuNPs. Au/TiO_2_ is also the most selective catalyst to semi-hydrogenation with 100% of butenes at 100% conversion and no significant amount of propane formed, in agreement with previous studies.^67^ In contrast, Ni/TiO_2_ is the most active sample, displaying the lowest *T*_100%_ (90 °C) and the least selective catalyst, it produced 14–15% of propane and butane at 100% conversion.

**Table 2:** Temperature and selectivities to products at 100% conversion of butadiene over Au(10)Ni/TiO_2_, Au(20)Ni/TiO_2_, Au(30)Ni/TiO_2_, Ni/TiO_2_ and Au/TiO_2_ as a reference

Catalysts	Catalytic bed			At 100% of butadiene conversion			
	Catalyst mass (mg)	Ni number (μmol)	Au number (μmol)	*T*_100%_ (°C)	Conversion to propane (%)	Selectivity to butane (%)	Selectivity to butenes (%)
Ni/TiO_2_	2.5	0.52	—	90	13.7	15.5	84.5
Au(10)Ni/TiO_2_	5	0.64	0.08	83	4.4	6.4	93.6
Au(20)Ni/TiO_2_	5	0.53	0.16	98	1.0	1.0	99.0
Au(30)Ni/TiO_2_	5	0.49	0.27	120	1.3	1.2	98.8
1 wt% Au/TiO_2_^a^	100	—	6.1	225	0.2	0	100

a From ref. 8.

After Au was added to Ni/TiO_2_, *T*_100%_ increased gradually with the Au loading, indicating a lower activity than Ni/TiO_2_ since there is almost the same amount of Ni in the catalytic bed. This lower activity can be explained by the lower amount of Ni exposed on the NP surface due to either the replacement of some Ni atoms by Au atoms on particle surfaces or the increase of the NP size (Fig. 1). The *T*_100%_ measured for Au(10)Ni/TiO_2_ sample, lower compared to Ni/TiO_2_, can be explained by the slightly higher number of Ni in this sample (0.64 μmol *versus* 0.52 μmol in Ni/TiO_2_ sample, Table 2). Concomitant to the gradual increase of *T*_100%_ with the Au loading, the selectivity to propane and butane drastically decreased, and that to butenes increased. The latter reached ∼99% for Au(20)Ni/TiO_2_ and Au(30)Ni/TiO_2_ while the formation of propane became negligible (∼1% conversion) at *T*_100%_. In other words, the AuNi/TiO_2_ catalysts became more selective to semi-hydrogenation than Ni/TiO_2_ as the gold loading increased. Moreover, the selectivity to butenes became similar to that of Au/TiO_2_ in spite of a much smaller amount of gold in the catalytic bed than in Au/TiO_2_ (0.16 to 0.27 μmol for Au(20)Ni/TiO_2_ and Au(30)Ni/TiO_2_, respectively, *versus* 6.1 μmol for Au/TiO_2_, see Table 2). Also, their activities were much higher than that of Au/TiO_2_ in spite of larger NPs (4.7 nm *versus* 2.7 nm, see Table 1), possibly because of the presence of Ni in the metal particles.

**Fig. 2:**
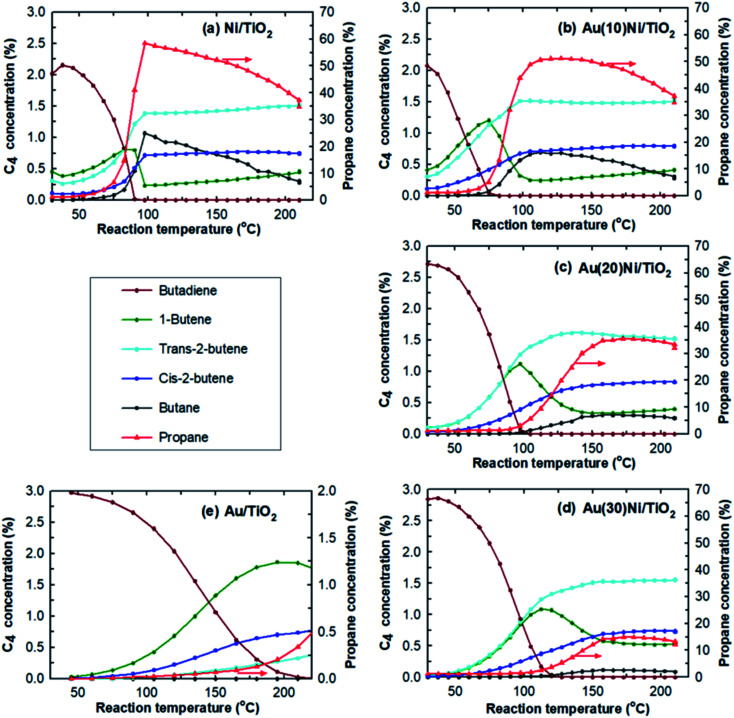
Evolution of the concentrations in butadiene and the different products as a function of the reaction temperature over (a) Ni/TiO_2_, (b) Au(10)Ni/TiO_2_, (c) Au(20)Ni/TiO_2_, (d) Au(30)Ni/TiO_2_ and (e) Au/TiO_2_ catalysts after *in situ* activation at 350 °C in H_2_.

The selectivities to the different butenes (1-butene, *trans*-2-butene and *cis*-2-butene) can now be closely examined (Fig. 2). Firstly, the comparison of the selectivities to the different butenes for the two monometallic Ni/TiO_2_ and Au/TiO_2_ catalysts showed that *trans*-2-butene, the thermodynamically stable butene, was the main isomer formed over Ni/TiO_2_ while 1-butene was the main isomer formed over Au/TiO_2_, in agreement with our previous study.^67^ Secondly, the comparison of the selectivities of the AuNi/TiO_2_ catalysts showed that when the Au percentage increased, the selectivity to butenes increased, while the order of the selectivities to different butenes remained roughly the same as that of Ni/TiO_2_. From all these results, one could deduce that the addition of small amount of gold to nickel allowed a drastic improvement of the selectivity to semi-hydrogenation while activity remains close to that of nickel in spite of the larger particle sizes in the AuNi/TiO_2_ catalysts, *i.e.* the lower metal surface area. This is clearly shown if one compares the catalytic results of Au(20)Ni/TiO_2_ with those of Ni/TiO_2_, two samples that contain the same amount of nickel, Au(20)Ni/TiO_2_ allowed for better selectivity (99% *versus* 84.5% for Ni/TiO_2_) while the two samples show close *T*_100%_ in spite of the larger average particle size of Au(20)Ni/TiO_2_ (4.2 nm *versus* 1.6 nm for Ni/TiO_2_).

These catalytic results suggest that Au and Ni are closely interacting, as it is further elaborated in the discussion part. To emphasize this point, a previous result from our group^8^ showed that a mechanical mixture (50–50% in catalyst weigh) of monometallic Au/TiO_2_ (1 wt%) and Ni/TiO_2_ (0.2 wt%), *i.e.*, corresponding to an Au/Ni atomic ratio of 1.7, exhibited the same catalytic behavior (activity and selectivity) as the monometallic Ni/TiO_2_ sample. These results also suggest that to understand how the catalytic properties are modified by the addition of gold, the metal particle microstructure must be known. To this end, we have performed a detailed investigation of individual nanoparticles by advanced electron microscopy techniques.

### Atomic structure of nanoparticles

**Fig. 3:**
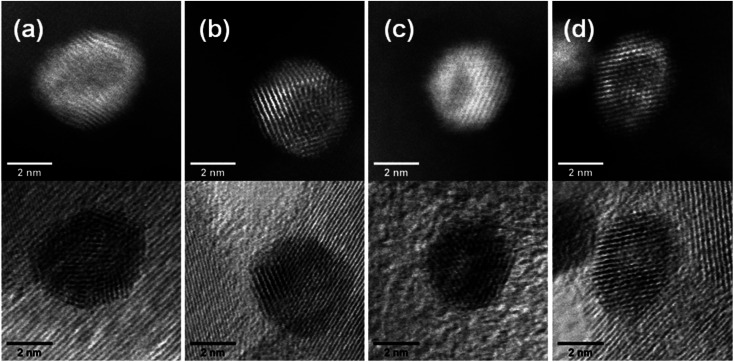
STEM images in both the dark field (HAADF) (top row) and the bright-field mode (bottom row) showing core–shell nanoparticles in: (a) as-prepared Au(10)Ni/TiO_2_, (b) Au(10)Ni/TiO_2_ after activation (H_2_ at 350 °C), (c) as-prepared Au(30)Ni/TiO_2_ and (d) Au(30)Ni/TiO_2_ after activation.

**Fig. 4:**
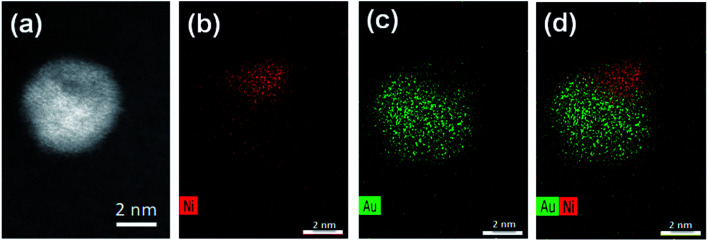
STEM and EDX mappings for an AuNi particle in Au(10)Ni/TiO_2_: (a) the STEM dark field image; (b) EDX mapping of Ni; (c) EDX mapping of Au; and (d) combined EDX mapping of Ni and Au.

**Fig. 5:**
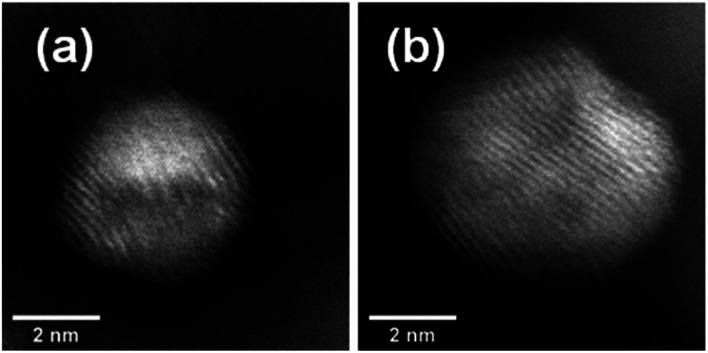
HAADF-STEM images showing (a) an off-centered core–shell nanoparticle and (b) a multi-centered core–shell nanoparticle, all from as-prepared Au(10)Ni/TiO_2_.

Aberration-corrected STEM images of nanoparticles in samples Au(10)Ni/TiO_2_ and Au(30)Ni/TiO_2_ are shown in Fig. 3 (see also Fig. S1‡). In both samples, whether imaged before (as-prepared) or after activation (350 °C under H_2_), the particles show a distinct contrast between a darker area near the center of the NPs and a lighter one near the surface (in the HAADF-STEM mode). Considering the large difference in atomic numbers between Au (*Z* = 79) and Ni (*Z* = 28), and the knowledge that HAADF-STEM intensity is roughly proportional to *Z*^1.7^,^68,69^ the data clearly suggested that the nanoparticles were composed of a core rich in nickel and a shell largely made of gold. This was also consistent with the EDX mapping performed on one of the particles, which showed that the part of the NP with darker image contrast was Ni-rich and that with a lighter image contrast was Au-rich (Fig. 4). In this particle, the Ni-rich region is also seen clearly off-centered, a common observation in our samples (see Fig. 5a and S1‡) and which had been predicted for bimetallic alloys with large lattice mismatch (∼16% between Au and Ni), poor miscibility and tendency of the bigger atoms to segregate close to the surface.^70^ Another type of core–shell structures was also noticed. One could find particles whose images, displaying multiple darker regions, suggesting multi-centered Ni cores surrounded by Au-rich shells (showing bright contrast) (Fig. 5b and S2‡). These particles with multi-centered Ni cores might arise from the coalescence of several smaller Ni cores. The set of images also show clearly that the structures of all nanoparticles examined do not depend on the amount of gold or whether the samples had been activated or not. Particles without large contrast variation were sometimes observed (Fig. S3‡), suggesting the existence of Au-rich nanoparticles. However, they are not further considered as they are not prevalent and cannot be responsible for the dominant catalytic effects we have observed.

## Discussion

The results of chemical analysis shown in Table 1 are consistent with the stoichiometric Ni replacement by Au according to eqn (2), and provide direct evidence that the GR reaction worked properly. This evidence is also supported by the formation of core–shell nanostructures in bimetallic nanoparticles imaged by STEM and EDX (Fig. 3–5, S1 and S2‡), where Ni is mainly located in the core of the particles, either as centered, off-centered or multi-centered core, while Au constitutes the main element of the outer shell. This is also consistent with the GRR process, during which the replacement of Ni atoms by Au atoms occurs at the surface of the Ni nanoparticles, resulting in an Au-enriched outer shell.

Regarding the thickness and composition of the shell, the GRR process would ideally result in a gold atomic monolayer, *i.e.* once the Ni on the surface has been replaced by gold, the GRR should stop. However, the detailed kinetics of GRR at the particle surfaces is unknown at the moment, and the STEM images show that a much thicker Au shell formed on the Ni NPs (Fig. 3). Similar observations were reported in the literature for other bimetallic nanoparticles prepared by GRR. Zhang *et al.*^54^ mentioned in a study on PdAg/SiO_2_ that the actual Pd deposition was beyond the theoretical limit of one atomic layer. The authors proposed that the large difference in Pd and Ag surface free energies resulted in Pd diffusion into the bulk of Ag particles or in Ag diffusion to the surface, which provided exposed Ag atoms for further galvanic displacement. This phenomenon was also observed by An *et al.*^47^ for AuAg NPs supported on Fe_2_O_3_–carbon composite. Other authors showed that GRR of Ag by Au could even lead to the formation of NPs consisting of hollow nanoshells made of AuAg alloy.^71,72^ Sun *et al.*^72^ proposed that the formation of these hollow nanoshells was the result of the combination of galvanic replacement reaction and alloying, leading to more thermodynamically stable structure than pure Au or Ag. All these observations suggested that the metal atoms of the core had the ability to diffuse within the shell during GRR, hence leading to the formation of an alloyed shell.

However, in contrast to the AuAg and PdAg systems cited above, Au and Ni are immiscible metals in their bulk-state.^14^ As mentioned in the introduction, according to the thermodynamic calculations published by Xiong *et al.*^15^ and the experiments performed by Bogatyrenko *et al.*,^16^ the miscibility gap (the composition range within which Ni and Au are not miscible) depends on the size of the bimetallic nanoparticles. At 300 K, the temperature at which the present study was carried out, the miscibility gap is reduced to a Ni composition range of 14–96% for a 13 nm size AuNi particle,^15^ and to 40–80% for a 6 nm size AuNi particle.^16^ For bimetallic nanoparticle below 4 nm in size, NiAu alloy formation is expected throughout the whole composition range, at thermal equilibrium.^15^

A closer examination of the images of EDX mapping shown in Fig. 4 revealed that a residual Ni signal may be seen within the Au-rich part of the nanoparticle and that inversely, a residual Au signal may be detected within the Ni-rich part of the nanoparticle. This suggests that the formation of a AuNi alloy is possible, to some extent, as in the case of the AuAg and PdAg systems. Alloying would result in Au shells containing a small proportion of Ni alloyed and *vice versa*, in Ni-cores containing a small proportion of Au alloyed. This is indirectly supported by lattice spacing analysis discussed below.

**Fig. 6:**
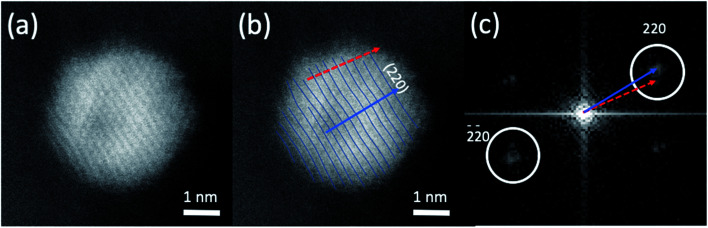
(a) A high resolution HAADF-STEM image of the atomic column structures of a bimetallic core–shell nanoparticle imaged approximately along the [111] axis. (b) The same image with one set of (220) atomic planes traced out. The dashed (red) and solid (blue) arrows are the normal directions of the atomic planes. (c) The Fourier analysis of the HAADF-STEM images with the dashed (red) and solid (blue) arrows indicating the splitting of the (220) ‘diffraction spots’ from the corresponding atomic planes shown in (b), due to distortion of the strained atomic planes. The value of 5% of the lattice-spacing difference could be measured from the arrowed vectors, which give a measure of the lattice spacings over the whole particle. Since the lattice spacings are not calibrated, only their ratio (expressed in %) could be determined. The difference in the orientation of the arrowed vectors in (c), also averaged over the whole particle, is related to the bending of the atomic planes already observed in (b).

The HAADF-STEM images of NPs closely aligned on high symmetry zone-axis orientation allow the resolution of the atomic columns or planes, indicating that the core–shell particles are crystallized (Fig. 3 and 5). They often display a coherent structure across the core–shell boundary. Fig. 6a represents an example of a particle imaged along its (111) zone axis with two independent sets of {220} planes. In Fig. 6b, one can see that the (220) atomic planes continue smoothly from one side of the nanoparticle to the other one, even when they pass through the area imaging the Ni-rich core. Interfacial dislocations, like the one observed on the left edge of the core in Fig. 6b, can be observed in some NPs, but they are rare. This observation demonstrates that the Au-rich shell largely forms epitaxially on the Ni-core. Fig. 6a also shows that the atomic planes slightly bend to accommodate larger atomic plane spacing at the particle surface, which indicates surface relaxation of coherently strained heteroepitaxy nanoparticles. The lattice spacing of a pure Au shell is expected to be 14% larger than that of a pure Ni core, according to the difference in their bulk lattice constants, *a*_Au_ = 0.408 nm and *a*_Ni_ = 0.352 nm. In our case, a much smaller value of the relative lattice-spacing difference, 5%, was measured. The value could be obtained directly from the HAADF-STEM image (Fig. 6a and b) or deduced from the relative change in the lengths of the dashed (red) and solid (blue) arrows in the Fourier transform of the HAADF-STEM image in Fig. 6c (see details in Fig. 6 caption), both giving similar result. This reduced difference between the average lattice spacing of the shell region and that of the interior of the nanoparticle is consistent with the non-trival core–shell model of the particles we proposed from the examination of Fig. 4, *i.e.*, the shell is not pure gold but Au-rich, and the core is not pure Ni but Ni-rich. Finally, the observation that the diffraction spot at the extremity of the blue arrow (surrounded by a white circle in Fig. 6c) is diffused, indicates spatial inhomogeneities of the strain inside the nanoparticle. Such a complex strain distribution is common in strained nanoparticles.^73^ In contrast, the diffraction spot as a small dot at the extremity of the red arrow indicates locally a relative homogeneity of strain in the corresponding part of the nanoparticle shell.

Based on all these observations, we thus believe that the strain, its inhomogeneities and the alloyed composition of both the core and the shell are two intertwined features of the surfaces of the (Ni-rich) core-(Au-rich) shell nanoparticles that make them different from nanoparticles with surfaces of either pure Ni or Au nanoparticles.

These two features may explain the catalytic results obtained with the AuNi/TiO_2_ catalysts. However, before discussing this point, it is worth to remind that the catalytic reaction takes place at temperatures between RT to 210 °C and mainly under H_2_ (20% H_2_, since propene (30%) does not react), and that the core–shell structure of the AuNi NPs is certainly preserved during reaction since we showed that the structure remained unchanged when the as-prepared samples were activated at 350 °C under H_2_ (Fig. 3). Hence, both the facts that the addition of small amount of gold to nickel drastically improved the selectivity of nickel to semi-hydrogenation while keeping the distribution of butenes similar to that of Ni/TiO_2_, and that the catalyst activity remained close to that of nickel in spite of the gold shell, are an indication that the particle shell cannot be composed of only pure gold with a lattice spacing similar to its Au bulk values. As a consequence, it can be proposed that Au acts as a dilutent for nickel and reduces the size of the Ni atoms ensembles exposed on the particle surface, which modifies the adsorption mode and strength of butadiene and butenes.^74^ However, one can wonder how a small proportion of Ni close to and/or on the gold shell surface of the AuNi particles, with sizes significantly larger than the initial Ni particles in Ni/TiO_2_ (Fig. 1), can provide a catalyst with activity close to that of Ni/TiO_2_. This is clearly an indication of a synergistic effect. The strain-induced effect due to epitaxial core–shell nanoparticle structure and the alloyed nature of both the core and shell could make the catalytic surface neither Ni- or Au-like and could be the physical basis for the observed synergistic effect, but more experiments or better theoretical simulations would be required to substantiate this point. Factors such as particle shape change due to misfit strains and surface energy of the alloyed surface should also be taken into consideration to give a more realistic description of the catalytic properties of the AuNi/TiO_2_ catalysts.

The significantly larger sizes of the AuNi particles after GRR than that of the original monometallic Ni ones (Fig. 1) in spite of the low average Au/Ni ratio (0.13 to 0.55) was unexpected. In principle, GRR leads to a decrease in the total number of atoms in particles because of the stoichiometry of the reaction (2Au atoms replace 3Ni atoms – eqn. (2)). However, because of the larger lattice spacing of bulk Au (0.408 nm) compared to that of Ni (0.352 nm), the atomic volume expansion factor can be as large as (0.408/0.352)^3^ = 1.55. As a result, the theoretical nanoparticle size ratio before and after GRR of a Ni particle is estimated to be 1.04 (=1.55 × ⅔). The experimental size ratio appears to be much lower (approximately equal to 0.4 for Au(10)Ni/TiO_2_: 1.6 nm for Ni/TiO_2_*versus* 3.7 nm for Au(10)Ni/TiO_2_ in Table 1). This excessive size increase during GRR is difficult to explain in view of the literature, in which the evolution of the NP size before and after GRR has not yet been properly discussed and no clear trends has been shown. Both a slight decrease^50^ or an increase^44,49^ in size compared to the initial monometallic particles were reported for the preparation by GRR of either colloidal or supported AuAg particles. As 3Ag atoms are replaced by one Au atom during GRR, much smaller nanoparticle sizes are expected. With regard to AuNi particles, there are very few cases in the literature of preparation of supported Au–Ni catalyst by GRR (as mentioned in the Introduction). Wu *et al.*^3^ synthesized AuNi NPs on TiO_2_ by GRR under experimental conditions close to ours but from much larger Ni nanoparticles (∼45 nm) than ours, and obtained AuNi NPs smaller than the starting Ni ones. They tentatively explained this unexpected result as due to partial dissolution of a Ni^0^ fraction under the acidic pH imposed by the use of HAuCl_4_. The decrease in size described in the latter study is therefore the opposite of that observed in the present work. In our case, the increase of the AuNi particle size obtained after GRR (Table 1) and the presence of multi-centered core particles (Fig. 5b and S2‡) suggest an additional phenomenon of coalescence of Ni cores or AuNi nanoparticles during GRR. To explain the tendency of ultrasmall Ni NPs (<2 nm) to aggregate even though they are supposed to be in strong interaction with the TiO_2_ surface, we propose that the galvanic replacement of the Ni atoms at the Ni–TiO_2_ interface promotes the mobility of the Ni NPs over the TiO_2_ surface. Weakening of the Ni/TiO_2_ interface strength, possibly also due to the NiAu alloy formation within the shells under formation, likely facilitates the diffusion of the NPs, leading to the interaction with their closest neighbors. Such a phenomenon would explain both the thick shell of the NPs after GRR and the formation of multi-centered core NPs. Noteworthy, similar multi-centered core particles were also observed in unsupported AuNi colloids prepared by GRR.^60^

One can therefore conclude that the reaction of galvanic replacement of Ni by Au took place. However, we speculate that another chemical process of nanoparticle coalescence occurred concomitantly during GR reaction, likely because the initial Ni NPs were very small and therefore unstable upon Au deposition. This resulted in the formation of at least two different types of Ni core–Au shell NPs (single and multi-centered Ni cores, Fig. 3–5) whose both core and shell could contain a low proportion of atoms of the other metal. Although the nanoparticles increased in size after GRR, they remained rather small (<5 nm) compared to those reported in the literature so far.

## Conclusion

In this article, we reported the preparation of bimetallic Au–Ni catalysts synthesized with different Au/Ni atomic ratios (0.13, 0.30, 0.55) by galvanic replacement reaction (GRR) of Ni metal atoms in Ni^0^/TiO_2_ by Au^3+^ ions in solution. This GRR preparation method allowed the formation of bimetallic NPs in spite of the lack of miscibility of the two metals in the bulk. The establishment of the GRR process was confirmed by elemental analysis that demonstrated the replacement of Ni atoms by Au atoms according to the stoichiometry of the reaction (eqn (2)). After GRR, all the Au atoms in solution were deposited and able to cover the surface of the Ni nanoparticles resulting in the formation of bimetallic particles displaying Ni-rich core and Au-rich shell structures with centered, off-centered and multi-centered Ni cores, according to HAADF-STEM characterization. The formation of these nanostructures together with the unexpected increase of the AuNi NPs size after GRR indicated that GRR occurred together with a coalescence phenomenon. The structures and sizes of the bimetallic NPs were preserved after the activation treatment at 350 °C in H_2_ performed before the catalytic reaction, and certainly also during the reaction of selective hydrogenation of 1,3-butadiene in the presence of an excess of propene performed between RT and 210 °C. During the reaction, the AuNi/TiO_2_ catalysts showed a much higher selectivity to butenes than Ni/TiO_2_ at 100% conversion of butadiene with a close activity. The selectivity to butenes was as good as that of Au/TiO_2_ catalysts, *i.e.*, close to 100%, but at a much lower temperature than Au/TiO_2_. The fact that the distribution into the three types of butenes remained much closer to that of Ni catalysts than Au catalysts, and that some papers showed that the miscibility gap decreased when Au–Ni forms nanoparticles, allowed us to propose that the shell of the AuNi particles was not pure gold, but contained a low amount of Ni alloyed to gold; this alloying behavior was attested by the smaller than expected difference between the lattice spacing in the surface relaxed layer of AuNi particles and that of the Ni-rich core. Both the alloyed surface and the residual surface strain could be the physical basis for the observed synergistic effect in the catalytic behavior of the nanoparticles. The present work highlights the importance of detailed microscopy studies in order to fully understand the catalytic behavior of the AuNi/TiO_2_ catalysts prepared by GRR and of bimetallic catalysts in general.

## Conflicts of interest

There are no conflicts to declare.

## Supplementary Material

NA-003-D0NA00617C-s001
