# Supplementary material for: Synthesis of small Ni-core–Au-shell catalytic nanoparticles on TiO2 by galvanic replacement reaction
Source: Nanoscale Adv. 2021 Jan 8;3(3):823–35. doi: 10.1039/d0na00617c (PMC9418773; doi:10.1039/d0na00617c)
Supplement: NA-003-D0NA00617C-s001 [file NA-003-D0NA00617C-s001.pdf]

Electronic Supplementary Material (ESI) for Nanoscale Advances.
This journal is © The Royal Society of Chemistry 2020

Supplementary information

Synthesis of Small Ni-core-Au-shell Catalytic Nanoparticles on TiO_2 by Galvanic Replacement Reaction

Julien Reboul[1], Z.Y. Li[2], Jun Yuan[3], Kazuki Nakatsuka[1,4], Masakazu Saito[1,5], Kohsuke Mori[4], Hiromi Yamashita[4], Yu Xia[2,6], Catherine Louis[1]*

[1] Sorbonne Université, CNRS, Laboratoire de Réactivité de Surface (LRS), F-75005, Paris, France.
[2] School of Physics and Astronomy, University of Birmingham, Birmingham B15 2TT, U.K.
[3] Department of Physics, University of York, Heslington, York YO10 5DD, U.K.
[4] Division of Materials and Manufacturing Science, Graduate School of Engineering, Osaka University, 2-1 Yamada-oka, Suita, Osaka 565-0871, Japan
[5] Department of Chemistry and Materials Science, National Institute of Technology, Gunma College 580 Toriba-machi, Maebashi, Gunma 371-8530, Japan
[6] Department of Materials Science and Engineering, Southern University of Science and Technology, Shenzhen, Guangdong, China

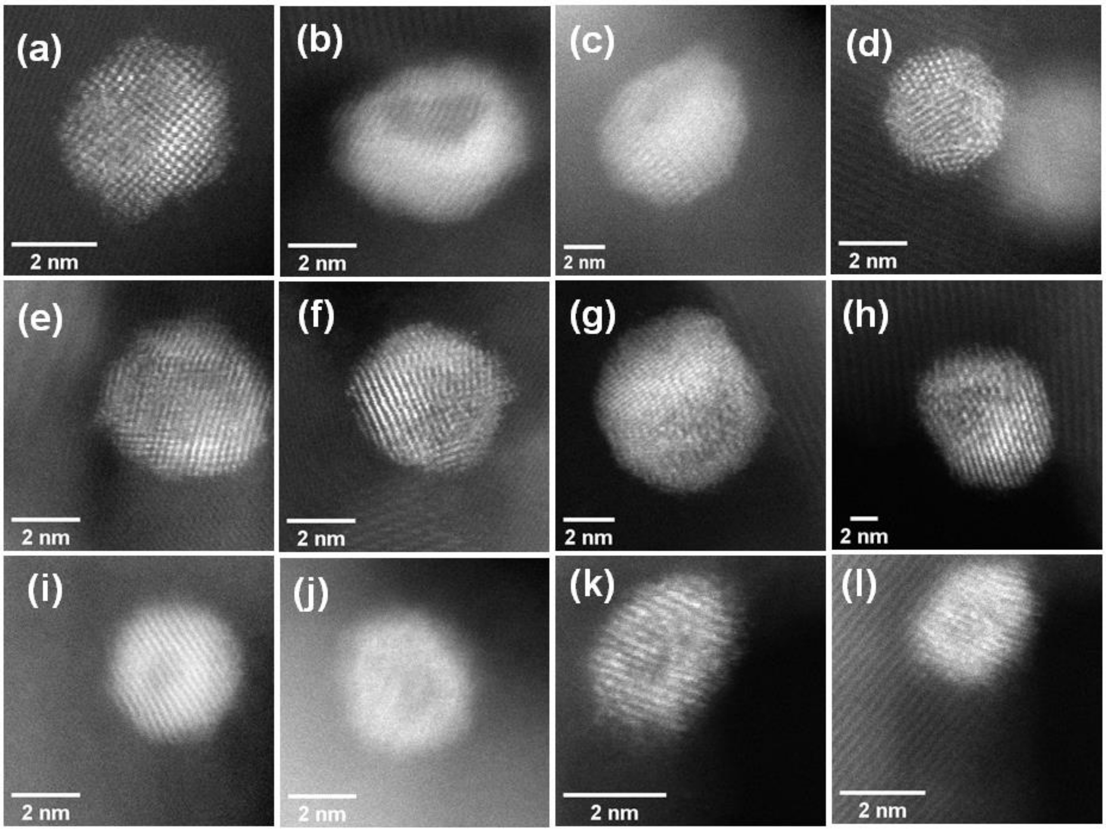

Figure SI-1 HAADF-STEM images, showing centered and off-centered core-shell nanoparticles, from Au(10)Ni/TiO_2 before (a-c) and after reduction (d-h); and Au(30)Ni/TiO_2 samples before (i) and after (j-l) reduction.

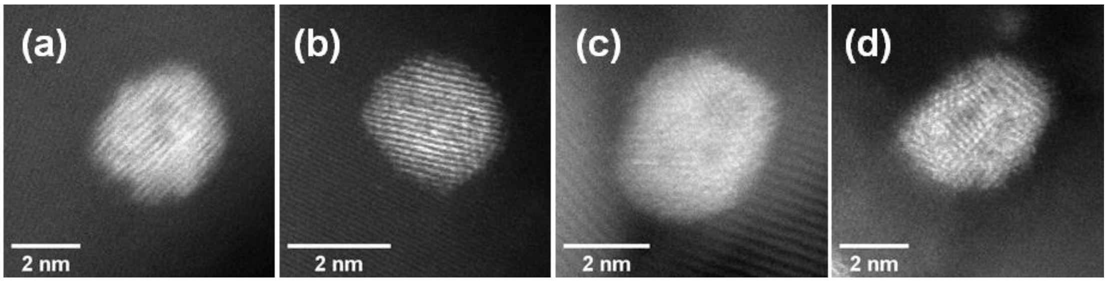

Figure SI-2 HAADF-STEM images, showing multi-cored nanoparticles, from Au(10)Ni/TiO_2 (a) as prepared; (b, c) reduced Au(10)Ni/TiO_2; and (d) reduced Au(30)Ni/TiO_2 samples.

SI-3: Among all the particles that we examined by electron microscopy, we also observed very few particles in AuNi/TiO_2 samples, showing no difference of contrast suggesting the formation of apparently pure Au nanoparticles (see **Figure SI-3**). However, one could not exclude the presence of a small percentage of nickel dissolved or segregated inside them [8].
However, to investigate whether gold particles could form as the result of mere Au adsorption on TiO_2, we performed the following experiment: A gold solution (6.17 µmol of Au, i.e., the equivalent Au loading of around 1 wt% Au, see Table 1) was added to TiO_2 first treated in the same conditions as for DPU but in the absence of Ni precursor. The suspension of TiO_2 in the gold solution had a pH ~3, i.e., a pH lower than PZC_{TiO2}, so the pH condition allowed $AuCl_4^-$ to interact with the positively charged TiO_2 surface. After washing with water and drying at RT under vacuum, the gold loadings were 0.9 wt%. The sample was colored after drying, indicating that at least part of gold was already reduced, probably because of TiO_2 (photoactive), and TEM showed the presence of both small (around 2-3 nm) and larger NPs (around 5-7 nm).
However, the following facts observed in the Au-Ni samples:
(i) the large majority of the particles observed by TEM are bimetallic (and the Au shells formed on the Ni NPs are often much thicker than expected as already mentioned on p. 14 and discussed on p. 18),
(ii) one cannot exclude the presence of small percentage on nickel in the seemingly pure Au nanoparticles
(iii) Table 1 shows that the Ni loading in AuNi/TiO_2 decreased when the Au loading increased and that the resulting Au/Ni atomic ratios corresponded to those expected according to Equation 2,
are an indication that the mechanism of GRR is predominant compared to Au adsorption.

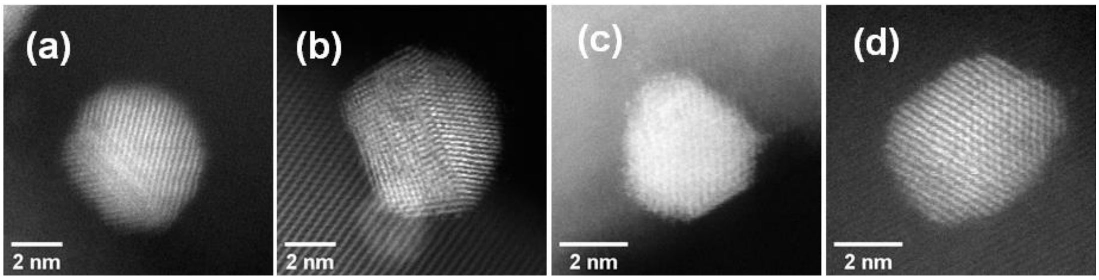

Figure SI-3 HAADF-STEM images of nanoparticles, from Au(10)Ni/TiO_2 (a,b) and Au(30)Ni/TiO_2 (c,d) samples before and after reduction. They showed no core-shell or multi-centered contrasts and are likely Au-only particles.
